# Supplementary material for: Removal of Fluoride from Aqueous Solution Using Shrimp Shell Residue as a Biosorbent after Astaxanthin Recovery
Source: Molecules. 2023 May 5;28(9):3897. doi: 10.3390/molecules28093897 (PMC10180352; doi:10.3390/molecules28093897)
Supplement: Supplementary file 1 [file molecules-28-03897-s001.zip › molecules-2348462-supplementary.pdf]

Supplementary material

Figure Captions

Figure S1. Micrographs of shrimp shells before and after dissolving in [Emim]Ac with solid-liquid ratio of 1:20 g/g at 100℃ for 2 h.

Figure S2. X-ray diffraction patterns for shrimp shells before and after astaxanthin recovery at different extraction conditions: (a) solid-solvent ratio at: size = 180-250 μm, T = 60 °C, and t = 2 h; (b) pretreatment time at: size = 180-250 μm, T = 60 °C, and solid-solvent ratio = 1:10; (c) size of shrimp shells at: solid-solvent ratio = 1:10, T = 60 °C, and t = 2 h; (d) pretreatment temperature at: solid-solvent ratio = 1:10, size = 180-250 μm, and t = 2 h.

Table Captions

Table S1. The thermodynamic parameters for fluoride adsorption with ETSS.

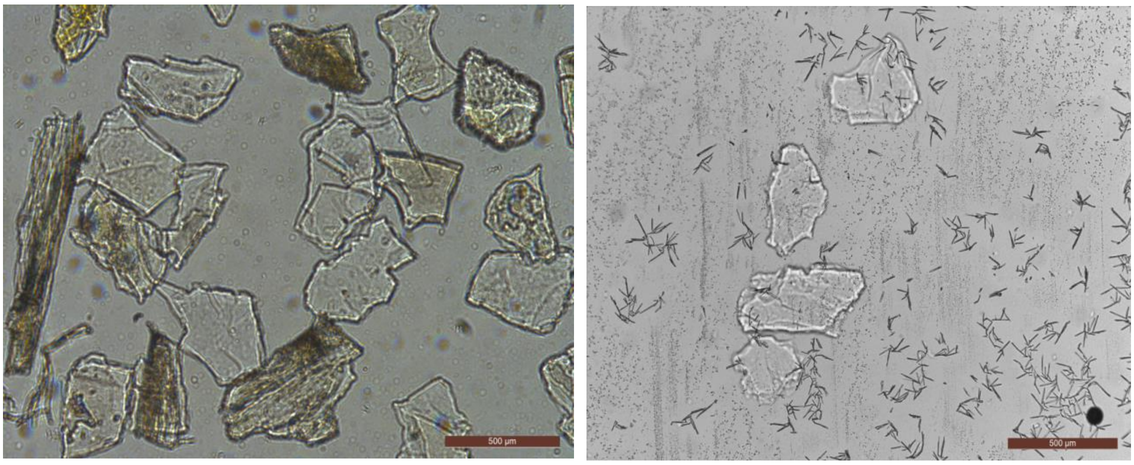

(a)Shrimp shells (b)Shrimp shells dissolved in [Emim]Ac

Figure S1.

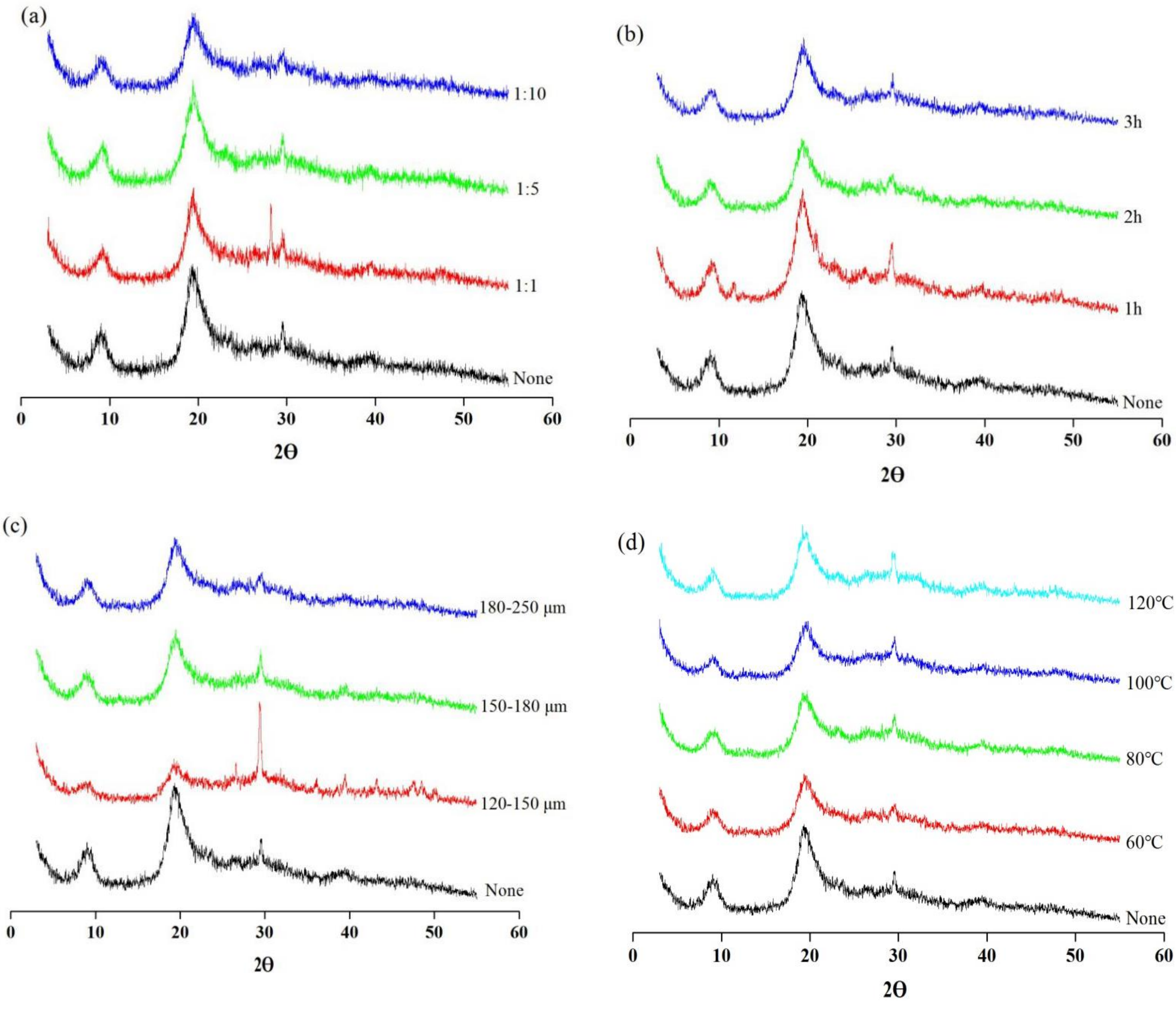

Figure S2.

Table S1

The thermodynamic parameters for fluoride adsorption with ETSS[a].

Tempeature (℃)	ΔG (kJ·mol^{-1})	ΔH^0 (kJ·mol^{-1})	ΔS^0 (kJ ·mol^{-1}· K^{-1})
20	5.20	-	-
30	4.50	-	-
40	4.39	9.58	0.02
50	4.43	-	-
60	4.49	-	-

[a] adsorbent dose = 10 g/L, C_0 = 50 mg/L, time = 8 h
